# Intra-aural tick bite causing unilateral facial nerve palsy in 29 cases over 16 years in Kandy, Sri Lanka: is rickettsial aetiology possible?

**DOI:** 10.1186/s12879-018-3338-8

**Published:** 2018-08-22

**Authors:** Senanayake A. M. Kularatne, Ranjan Fernando, Sinnadurai Selvaratnam, Chandrasiri Narampanawa, Kosala Weerakoon, Sujanthe Wickramasinghe, Manoji Pathirage, Vajira Weerasinghe, Anura Bandara, Jayanthe Rajapakse

**Affiliations:** 1 Department of Medicine, Faculty of Medicine, University of Peradeniya, Peradeniya, 20400 Sri Lanka; 2 ENT Unit, General Hospital, Kandy, 20000 Sri Lanka; 3 Department of Parasitology, Faculty of Medicine and Allied Sciences, Rajarata University of Sri Lanka, Saliyapura, 50008 Sri Lanka; 4 Department of Physiology, Faculty of Medicine, University of Peradeniya, Peradeniya, 20400 Sri Lanka; 5 Department of Veterinary Pathobiology, Faculty of Veterinary Medicine and Animal Science, University of Peradeniya, Peradeniya, 20400 Sri Lanka

**Keywords:** Rickettsial, Facial nerve palsy, Tick bite, Otoacariasis, Sri Lanka

## Abstract

**Background:**

Over the last two decades intra-aural tick infestation (otoacariasis) has been a common occurrence in the hilly central region in Sri Lanka. Very occasional detection of isolated unilateral facial nerve palsy associated with otoacariasis attributed to toxin damage of the nerve prompted us to study the clinico-epidemiology and aetio-pathology of the problem.

**Methods:**

All cases having isolated unilateral facial nerve palsy associated with otoacariasis presented to, Ear Nose and Throat clinic at General Hospital Kandy, Sri Lanka from 2001 to 2016 were included in the study. The facial palsies were assessed with nerve conduction studies and, harvested ticks were identified.

**Results:**

There were 29 patients with mean age of 46 years (range 22–76 years) with male to female ratio of 1:1.9. First 12 patients without specific treatment took 1–55 months for recovery and 4 had axonal degeneration. Last 5 patients were treated with doxycycline and recovered in 4 weeks. They had strong sero-conversion of immunofluorescence antibodies against spotted fever rickettsioses and the tick harvested from the last patient was PCR positive for rickettsial DNA. Identified ticks belonged to *Dermacentor*, *Amblyomma*, *Rhipicephalus* and *Hyalomma* species.

**Conclusions:**

On contrary to popular toxin theory, we were able to demonstrate treatable rickettsial aetio-pathology as the cause of otoacariasis associated lower motor facial palsy in Sri Lanka.

## Background

**Fig. 1:**
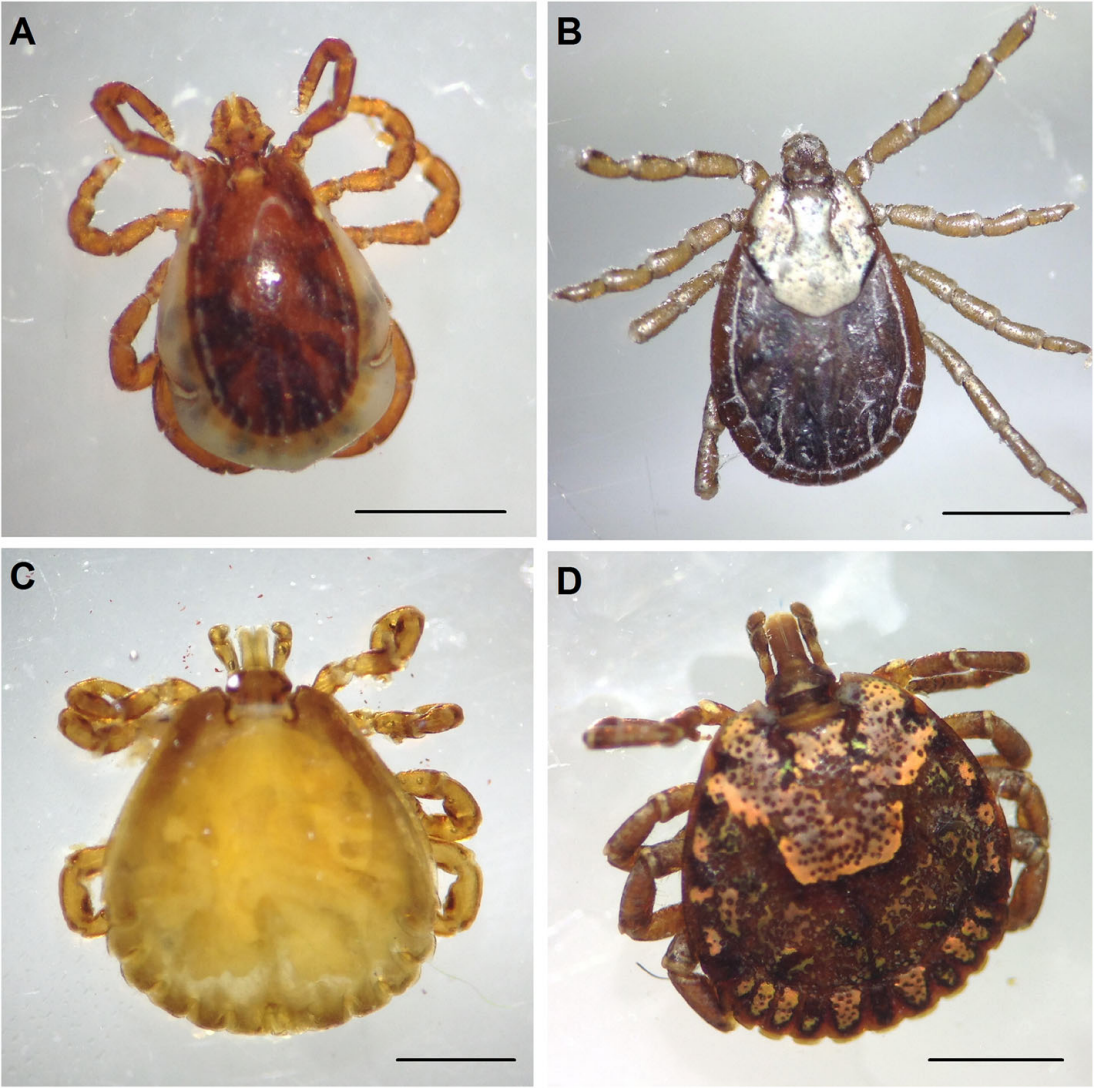
Dorsal view of ticks. **a** *Rhipicephalus* adult female, **b** *Dermacentor* adult female, **c** *Hyalomma* adult female, **d** *Amblyomma* adult female. Scale bars 1 mm

Sri Lanka, a tropical island in the Indian ocean has a rich biodiversity including its ubiquitous tick population which comprises about 31 species of ticks belonging to 11 genera [1–4]. Ticks belong to genera of *Dermacentor*, *Amblyomma*, *Hyalomma*, *Boophilus* and *Rhipicephalus* are common vectors of both animal and human diseases in Sri Lanka [1, 4] Ticks have three stages of development namely larva, nymph and adult each of which is capable of transmission of diseases. The tick infestation of ear canal is called otoacariasis. Over the last two decades intra-aural tick infestations are becoming a common problem seen at the ear-nose-throat (ENT) clinics in the central hills of Sri Lanka, however, the exact incidence remains unknown. Reports of tick infestations in humans are few in Sri Lanka, first being a report published in 1965 describing 4 genera of ticks recovered from human skin [2, 3]. At the dawn of the century, first report of intra-aural tick infestations was published in 2003 from the ENT clinic, General hospital, Kandy, Sri Lanka where investigators described 29 patients and recovery of nymphs of 29 *Dermacentor auratus* and one *Hyalomma marginatus isaaci* from ear canals of patients [5]. From the same clinic another publication describes 66 patients with otoacariasis in 2014 [6]. Another paper from Ratnapura district of Sri Lanka described 870 patients of intra-aural tick infestation over 2 years from year 2000 where most of the offending ticks belonged to genera of *Rhipicephalus, Amblyomma* and *Hyalomma* [7] (Fig. 1). Even though, intra-aural tick infestation is an increasing problem, virtually all patients make recovery without local or systemic clinical problems. However, by 1999, a very occasional detection of unilateral facial palsy associated with intra-aural tick bite at the ENT clinic, Kandy aroused curiosity and prompted us to investigate such cases from 2001. As it was an established fact that some tick species are capable of producing paralytic toxins [8], we attributed tick toxicosis and paralysis to explain the emerging problem of 7th cranial nerve palsy. Thus patients were managed as if other cases of facial palsy [9], but recovery was slow and some patients took years. However, considering the sporadic nature of the cases of facial palsy despite high incidence of otoacariasis, we thought about other possible aetio-pathological conditions by about 2011. Based on the fact that ticks are known vectors of numerous pathogens, e.g. *Rickettsia*, *Borrelia* and *Coxiella* [9], we decided to investigate our patients to find out such association particularly with spotted fever rickettsioses. This hypothesis was further supported by the knowledge of lyme neuroborreliosis caused by *Borrelia burgdorferi* which is transmitted by certain ticks of the genus *Ixodes* [10–13]. Furthermore, we noticed a parallel phenomenon of emergence of tick borne rickettsial infections in the same region in late nineties in the central hilly provinces in Sri Lanka [14, 15]. As there were no records of intra-aural ticks with facial palsy in eighties and nineties we considered this as an emerging condition linked to rickettsial aetiology more than tick toxins. The aims of this study were to analyze clinical records of the patients, to follow up the recovery with nerve conduction studies, to investigate to ascertain rickettsial aetio-pathology, and to document recovery with anti-rickettsial antibiotics.

## Methods

### Patients and settings

The ENT clinic, Kandy is a tertiary referral center for management of otoacariasis where this study was carried out from 2001 to 2016 In difficult cases, ticks are removed under general anesthesia. But in most cases, the affected ear is instilled with 25% dextrose in glycerin and left for about 2 weeks to kill the tick and is removed with its mouth parts. Those who developed associated facial nerve palsy were admitted to Teaching Hospital, Kandy and subsequently transferred to Teaching Hospital, Peradeniya (THP) for further management. At THP, patients were assessed and clinical data were recorded. All patients underwent nerve conduction studies (NCS) to assess the severity of the nerve palsy, and NCS were repeated periodically to assess the recovery. Electromyography was performed using concentric needle electrodes. Symptomatic medications were given and patients were referred for regular physiotherapy. The respective specimens of ticks were collected for identification and further testing.

Between 2001 to 2011 we did not attempt to make aetiological diagnosis believing tick toxin as the cause of paralysis. However, in 2011 we changed our thinking pattern and decided to find out an alternative aetio-pathology of the disease. Thus in 2011, blood samples from newly admitted patients were tested for anti-rickettsial antibodies by indirect immunofluorescence test (IFT). Having established molecular diagnostic facilities, from 2015, molecular analysis of tick samples was done using a *Rickettsia* specific real time quantitative polymerase chain reaction (qPCR) assay.

Ticks, surgically-removed from the external ear canal of patients, were preserved and stored with alcohol in labelled vials. Identification of ticks was done by an expert in the field using standard keys and descriptions [2, 3, 16, 17]. The analysis of clinical data was done and presented in three periods. In first period, patients received routine management and had follow up for 6 years. In second period, acyclovir was included in the treatment regimen and follow up was incomplete. In third phase, we actively investigated patients for rickettsial aetiology.

### Serological analysis

Serological assessment was done with IFT to detect antibodies against *Rickettsia conorii* of spotted fever group using *R. conorii* IgG antibody kit (RCG-120; Fuller laboratories, California, USA). The assay utilizes cell culture-propagated *Rickettsia conorii* (Moroccan strain) as the substrate antigen and the assay was carried out according to the manufacturer protocol. Final sero-diagnosis of rickettsial infection was defined on the basis of the presence of a titre of 1/64.

### Molecular analysis

#### DNA extraction

DNA extraction from ticks were done as described previously [18]. In brief, individual adult ticks were cut longitudinally into halves using a clean stainless steel No.10 surgical blade (Swan- Morton, Sheffield, United Kingdom). Halved adult ticks were rinsed with distilled water then dried with Kimwipes paper (Kimberly Clark Worldwide Inc., Roswell, GA) prior to DNA extraction. Halved adult ticks were separately crushed in lysis buffer (buffer ATL) with proteinase K and incubated at 56 °C up to 48 h until the ticks were completely lysed. DNA was extracted using QIAmp DNA Mini Kit (QIAgen, Valencia, CA), according to the manufacturer’s instructions. DNA extraction of human whole blood samples were done by QIAmp DNA Mini Kit (QIAgen, Valencia, CA), according to the manufacturer’s instructions. The DNA of tick and human blood samples were stored at -20 °C until further use.

#### PCR analysis

qPCR was performed as previously published [18, 19] to detect *Rickettsia* using genus-specific 17RPA primers (17RPA-F: AGTAGGTGTAGGYGCATTACTT, 17RPA-R GAGTGTAYTCACGGCAATATTGA), for detection of Rickettsia genus-specific 17-kDa outer membrane antigen. Each reaction mixture of qPCR consisted of 10 ml of 2X RT2 SYBR Green qPCR master mix (4,309,155; SYBR® Green PCR Master Mix, Applied Biosystems®, Foster City, CA) 0.5 mM each of the forward and reverse primers (0.1 ml each), molecular biology grade water (7.8 ml), 2 ml of sample DNA template, or DNA-free template (water for negative control). Additionally, ROX passive reference dye (Thermo 75,768) 500 nM added to remove the background absorbent. The QuantStudio 6 Flex Real-Time PCR System (Applied Biosystems®, California, USA) was used to perform qPCR reactions and to analyze the results. qPCR cycling conditions were as follows: an initial holding stage 5 min at 95 °C followed with 40 cycles of denaturation at 95 °C for 30 s and annealing at 60 °C for 1 min. No DNA template controls were used in all the assays, as were positive controls.

## Results

Of the consecutive series of 29 patients with acute onset unilateral lower motor facial nerve palsy, the first case was recruited for the study in August 2001 and the 29th case in October 2016. The results are presented in three phases (periods) in a time scale to highlight the changing thinking pattern to find the aetio-pathology of the disease. The first phase includes 12 cases presented from 2001 to 2007. The second phase includes 6 patients covering the period from 2007 to 2009. The third phase includes 11 patients presenting from 2011 to 2016 (Table 1).

**Table 1:** Descriptive data of 29 patients with lower motor facial palsy due to intra-aural tick infestation over 15 years (Described 3 time periods; 1st to 3rd phase)

Descriptive data	1st Phase (2001–07)	2nd Phase (2008–09)	3rd Phase (2011 to 16)
Total number of patients	12	6	11
Mean age (range) years	45 (19–76)	50 (37–72)	49 (33–68)
Gender			
Male	6	3	1
Female	6	3	10
Tick infested ear and facial palsy			
Right	4	2	5
Left	6	4	1
No. of patients with otalgia	12	6	6
No. of patient with ear discharge	6	2	1
No. of patients with affected hearing	5	2	1
Mean duration of pain in the ear till detection of tick (range) days	5 (2–13)	3 (1–7)	4 (1–7)
Mean duration of time from pain in the ear to onset of facial palsy	7 (1–17)	5 (2–7)	5 (2–11)
Day of removal of tick from detection			
No. on same day	9	2	3
Delayed (day range)	1–3	1–9	1–5
No. developed palsy tick in-situ	8	3	4
No. developed palsy after removal of tick	4	3	7
Time range taken for onset of palsy after removal of tick	2–7	3	1–7
No. with neuropraxia	8	5	9
No. with axonal degeneration	4	1	2
Time for clinical recovery (range) in months	1–55	1–3	1–13
No. with partial recovery	2	2	2

The mean age of the whole case series is 46 years (range: 22–76 years) and comprises 19 females with male to female ratio of 1:1.9. The left ear is the most commonly affected side where intra-aural ticks were found in 18 (62%) patients. The rest were in the right ear canal. A tick per patient was found attached deep in the external auditory canal engorged with blood meal at the time of detection. Pain in the affected ear was the presenting complain in 27 (93%) patients with mean duration of pain of 5 days (range: 1–13 days). One patient presented with tinnitus of the affected ear for 2 days and other one had reduced hearing at presentation. Among all, 8 (26%) patients had hearing impairment and 9 (31%) patients had ear discharge of muco-purulent or blood stained fluid. These patients had no idea how they got the tick infestation and virtually all had domestic pets such as dogs or cats. In many situations, they reported the presence of wild animals such as monkeys, squirrels and night roaming pigs in their home garden and backyards. In the whole series, 16 (55%) patients developed lower motor facial palsy of the affected side while tick in-situ the ear, whereas 13 (45%) patients developed facial palsy days after the removal of tick from the ear canal with the mode of day 3 (range 1–7 days).

### 1st phase: From 2001 to 2007

The cohort comprises 12 patients with mean age of 45 years (range: 19–76 years) with equal gender distribution. Once detected, the ticks were removed on the same day in 9 patients and in the rest removal done within 1–3 days after detection. Eight patients developed lower motor facial nerve palsy of the affected side before removal of the tick whilst 4 patients developed the palsy 2–7 days after the removal of tick from external ear canal. No specific treatment was given for patients contemplating possible toxin mediated neuronal damage according to the popular belief that some species of tick excrete neuro- toxin in their saliva. However, they were given a short course of prednisolone to reduce inflammation, co-amoxyclav to cover secondary infections and continuous physiotherapy. They were followed up periodically with repeat nerve conduction studies. The complete clinical recovery took variable time period ranging from 1 month to 55 months. Of them, 4 patients took 12–55 months for recovery and they all had axonal degeneration. Of the offending ticks, 8 ticks were available without damaged head and body, and were identified as *Dermacentor auratus.*

### 2nd phase: From 2008 to 2009

This phase comprises 6 patients with the mean age of 50 years (range: 37–72 years) with equal gender involvement. In 2 patients, offending ticks were removed on the same day of detection and in 3 patients on 3rd day after detection, and in one patient it had taken 9 days for removal after detection. Three patients developed facial palsy while a tick attached in ear canal, however 3 patients developed the palsy on 3rd day after the removal of ticks. These patients were managed same as the former group of patients. Additionally, a short course of acyclovir was given empirically. At the end of 3 months 4 patients gained full recovery, among them one had axonal degeneration and others had neuropraxia. Two patients had partial recovery despite neuropraxia at 3 months. Unfortunately, they did not come back for follow up.

### 3rd phase: From 2011 to 2016

This phase comprises 11 patients with mean age of 49 years (range: 33–68 years) and 10 of them were females. Five patients developed facial nerve palsy before removal of tick from the ear canal and 6 patients developed facial nerve palsy 1–7 days after the removal of tick. In 2011, aetio-pathology of this condition was revisited and the treatments and investigations were directed towards rickettsial aetiology of this obscure condition. In addition to routine management, anti-rickettsial antibiotic doxycycline was given to 5 patients who presented in 2011 and 2014. However, the commencement of doxycycline in first four patients took time about 4 days after removal of tick and development of facial palsy. These four patients were followed up and they took 6 and 13 months respectively for full recovery. Their acute blood samples were tested for anti-rickettsial antibodies using IFT against *Rickettsia conorii* antigens and both were positive for IgG 1/32 titre level. However, further serology was not done for convalescent sera. The 5th patient was treated with doxycycline just after the development of facial palsy and she made full recovery in 1 month.

In 2015, we intensified the treatments and investigation to pursue the rickettsial aetiology. There were 4 patients in the year and three were treated with doxycycline just after removal of tick and the development of facial palsy (Table 2). They had neuropraxia in nerve conduction studies and recovered fully within 3 weeks. In all patients, acute sera were negative for IFA- IgG of *Rickettsia conorii*, but in convalescent sera IgG titres were 1/1024 confirming a strong seroconversion. The 4th patient of the year was a 33-year-old pregnant mother of 23 weeks of gestation. She presented with a history of pain in right ear of 3 days duration and developed right lower motor facial palsy. An engorged tick was found deep in the external auditory canal and was removed on the same day. Considering safety in pregnancy, she was treated with azithromycin which has anti-rickettsial properties. NCS revealed neuropraxia, and she recovered in 3 weeks. Anti-rickettsial IgG titre of her convalescent sera was 1/1024. Three offending tick specimen were available from these patients for identification and further studies. These ticks were adult stages of *Dermacentor*, *Hyalomma* and *Amblyomma* (Fig. 1). They were tested with a *Rickettsia* specific PCR assay and were negative for rickettsial DNA .

**Table 2:** Details of five patients who presented in 2015/16 and underwent investigations to find out aetio-pathology, treatment given and response to treatment

Description	Patient number				
	1	2	3	4	5
Month/ year of admission	3/15	4/15	5/15	6/15	11/16
Age –years	51	35	62	33	46
Gender/physical state	Female	Female	Female	Pregnant	Female
Affected ear and palsy	Left	Left	Left	Right	Left
Time for onset of pain to facial palsy-days	3	6	11	2	5
Time gap between removal of tick to onset of palsy in days (pre or post)	2-post	1 -pre	7-post	1-pre	0
Neuropraxia of facial nerve	Present	Present	Present	Present	Present
Treatment	Doxycycline	Doxycycline	Doxycycline	Azithromycin	Doxycycline
Time for full recovery in weeks	4	3	4	3	4
IFA – IgG tire for *R. conori* – acute sera	negative	negative	1/128	1/32	1/512
IFA – IgG tire for *R. conori* – convalescent sera	1/1024	1/1024	1/1024	1/1024	1/1024
Identification of tick	–	*Amblioma*	*Dermocentrer*	*Hyaloma*	*Rhipicephalus*
Tick for PCR-rickettsial DNA			Negative	Negative	positive

**Fig. 2:**
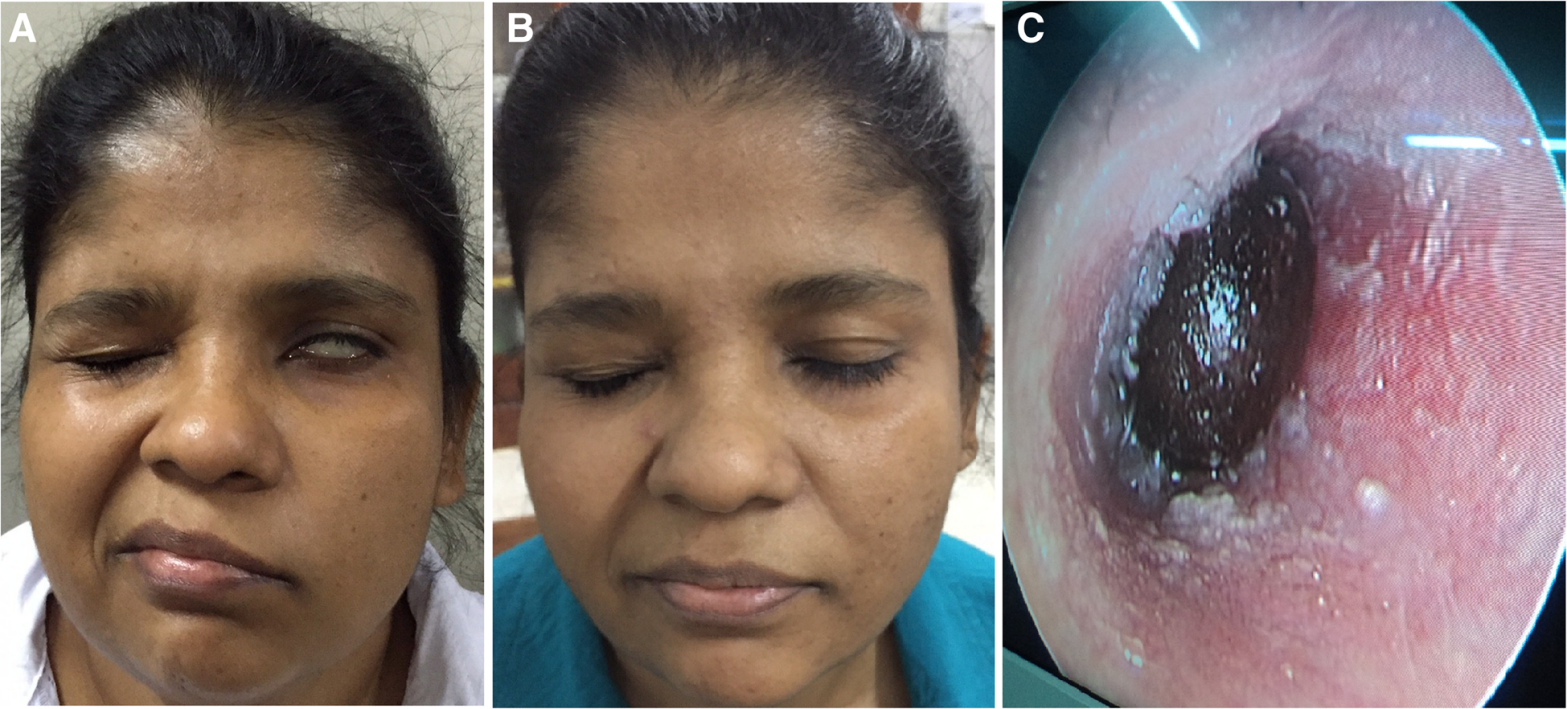
Intra-aural tick bite causing unilateral facial nerve palsy. Facial palsy of case 29, before (**a**) and after (**b**) treatment, **c** Tick attached to deep ear canal

The last patient of the series is a 46-year-old female presented in November 2016. She gave a history of pain in left ear of 5 days and development of facial asymmetry gradually on 5th day of pain. On the same day, the tick was removed after instillation of glycerin for few hours. On examination she had dense lower motor facial nerve palsy in the left side. The NCS revealed neuropraxia. She was treated with doxycycline 100 mg for 7 days and prednisolone 30 mg for 7 days and regular physiotherapy to affected side. She made full recovery in 4 weeks (Fig. 2). Her acute sera yielded IgG titre of 1/512 and convalescent sera showed a rising titre. Harvested tick from the ear drum was identified as *Rhipicephalus*, despite damaged head (Fig. 2). Further, testing of offending tick extract by real time PCR was positive for rickettsial DNA confirming the tick as the vector carrying the rickettsia. Simultaneously, patient’s serum was also was qPCR positive for rickettsial DNA.

## Discussion

Unilateral lower motor facial palsy has a myriad of differential diagnoses with the commonest being Bell’s palsy [9, 20]. We found strong association of otoacariasis with lower motor facial palsy of the same side of the face in 29 cases over 16 years from 2001. Despite high incidence of otoacariasis in the country, facial palsy occurred sporadically, with females and left ear being affected more. Pain and discomfort in ear were the commonest symptoms that preceded the palsy. Patients developed facial palsy whilst tick was in-situ or days after removal of ticks. During first phase of the study (12 patients), we believed on the theory of tick toxins as the causation of facial palsy and managed them symptomatically. Of them, 4 patients had axonal degeneration and had residual damage lasting for years. During the second phase study, we tried to hasten the recovery by giving acyclovir to the patients based on theory of possible herpes as in Bell’s palsy without much success. However, from third phase of the study since 2011, a rickettsial aetiology was postulated and commenced investigations and treatment. During this phase, patients presented until 2014 had positive IFT titre against spotted fever rickettsia and they recovered from facial palsy early with anti-rickettsial antibiotic doxycycline which was started with some delay. This observation encouraged us to intensify investigations of patients presented in 2015 (Table 2) where we observed seroconversion and rising titre of IFT antibodies against spotted fever rickettsioses (*R. conorii*). These patients were treated with doxycycline (azithromycin was given to a pregnant mother) at the outset of facial palsy and they made recovery in 3 weeks. In 2016, from the last patient in the series, we harvested a *Rhipicephalus* tick which gave positive result of PCR test for rickettsial DNA, testifying possible rickettsial aetio-pathology. In this study we found 4 species of ticks associated with facial palsy -*Dermacentor*, *Amblyomma*, *Rhipicephalus* and *Hyalomma*- which were the frequently encountered ticks in otoacariasis in Sri Lanka [4, 6, 7].

Intra-aural tick causing facial palsy was reported in 1996 in a 65-year-old Malay woman who made marked improvement of facial weakness following removal of *Dermacentor* tick attached to tympanic membrane [21]. Thereafter, few case reports are available reporting isolated cases of intra-aural tick causing facial palsy from Australia, Turkey and India [22–25]. In these cases, the offending tick species were *Ixodes holocyclus* in Australia and the hard tick *Hyalomma marginatum* from Turkey [22, 23, 25]. A 3-year-old child from India found to have left sided lower motor facial palsy and tick close to tympanic membrane which was removed under anesthesia. Upon removal of tick (unknown species) patient had recovered in 7 days [24]. Two patients in Turkey were adults and underwent removal of tick including mouth parts upon detection with a help of cup forceps apparently they recovered after 2 weeks [23, 25]. A case of right sided partial palsy was reported from an Australian traveler in Singapore who had a *Ixodes holocyclus* bite in right temporal scalp area [26]. In above reports, the authors reiterated the toxin theory to explain facial nerve palsy. The possibility of diffusion of toxin in tick saliva either in intact or preferably perforated tympanic membrane exposes facial nerve in fallopian canal which already gone through natural dehiscence [8, 25]. None of our patient had perforation of tympanic membrane, but offending ticks were found deep in the ear canal and they were removed after instillation of dextrose/glycerin and leaving days for ticks to die.

Tick paralysis is a well-known condition in both animals and man. This was first recorded in 1824 in Melbourne and its medical significance in humans was first recorded in 1912 in both Canada and Australia [8]. In general ticks are arachnids which include two families -*Argasidae* (soft ticks) and *Ixodidae* (hard ticks)- comprising more than 800 species. Of these, some 43 tick species in 10 genera are incriminated as causing tick paralysis in humans and animals [8, 27]. There is a difference of natural history of tick paralysis between North America and Australia. In North America, causative ticks are *Dermacentor andersoni* and *Dermacentor variabilis* which produce rapid muscle paralysis, also recovery is fast upon removal of ticks from the body. However, in Australia, *Ixodes holocyclus* commonly is commonly involved and paralysis tends to progress despite removal of ticks and last longer for days [8, 28]. Multiple haemostatic and anti-inflammatory agents are secreted from the salivary glands of tick, which facilitate attachment and feeding blood from host. These salivary glands are also capable of producing toxins that cause paralysis. Experimentally these toxins had been purified and even used for development of anti-toxins [8, 29].

Animal experiments using dogs, lamb and woodchucks in early days had suggested blockade at neuromuscular junction in tick paralysis [29, 30]. Further experiments suggested failure of acetylcholine release at the neuromuscular junction as the pathology in tick paralysis [31]. With the advent of neurophysiological investigations neuromuscular junction was reaffirmed as the site of the paralysis [8, 28]. However, normal motor conduction velocity with low amplitude compound muscle action potentials were revealed in Australian tick paralysis [8]. Tick paralysis in animals exists in Sri Lanka [1], but there are no records of humans cases reported in the country. It is not uncommon to see Guillain-Barre syndrome like cases in clinical practice, however, we cannot rule out overlooking cases of tick paralysis in such scenarios.

The fact that a significant number of cases developed facial palsy days after removal of intra-aural ticks and recovery taking long time in the first 12 patients in the series argue against the toxin mediated paralysis. We found both neuropraxia and axonal degeneration in neurophysiological studies in our patients which is contrary to the known patho-physiology of tick paralysis where transient neuro-muscular blockade occur at neuro-muscular junction. If toxin theory is true, toxins need to diffuse through the middle ear to reach facial nerve which takes its pathway in the medial wall of the middle ear in the facial canal. All our patients had intact ear drum that certainly prevent the direct access of tick saliva to middle ear. Stone et al. (1988) [32] suggested tick neurotoxin is a protein with molecular weight of 40,000 to 80,000 Da. As neuro-toxins are highly diffusible, it is unlikely to cause isolated nerve palsy rather than causing generalized paralysis.

Ticks are the reputed vectors for much life threatening infection in man and animal. These human infections include babesiosis (protozoa), spotted fever (rickettsiae),Q-fever (*Coxiella burnetii*), Lyme disease, relapsing fever (spirochete), viral infection such as Russian spring-summer encephalitis, Colorado tick fever, Crimean-Congo haemorrhagic fever [8, 33]. Some of these pathogens exhibits neurotrophism and able to produce diverse clinical manifestations. Among them, Lyme disease causes facial palsy due to infection with *Borrelia burgdorferi* which is transmitted by *Ixodes* ticks [10–12, 34, 35]. This is a treatable condition and responds well to doxycycline given over 21 days [20, 35]. Rickettsiae in general are neurotropic pathogens [36], but commonly cause vasculitis as the primary pathology [37, 38]. There are reports of cranial nerve palsies caused by rickettsial infections that support our hypothesis [36, 39–41]. History of rickettsial infections in Sri Lanka has two demarcated eras [42]. During the latter part of British colonial ruling (pre 1948) and during second world war, scrub typhus fever had been reported from Sri Lanka [43]. Since then, it was a silent period up to late nineties, that there were hardly any reports of rickettsial infection in Sri Lanka. However, dawn of new centaury reported emergence of rickettsial infection in central hills of Sri Lanka where we studied this case series of facial palsy [14]. Subsequent studies proved increasing burden of spotted fever rickettsioses in the region with diverse clinical manifestations including atypical neurological manifestations [15, 44]. Spotted fever rickettsioses are transmitted by ticks and it appears that tick populations harboring rickettsial pathogens are increasing in the region over last two decades. This could be attributed to changes in ecology and increasing number of wild animals (pigs, monkeys, rodents) and peri-domestic animals [45] in the region due to multiple reasons such as environment conservation laws and religious beliefs of people. In the backdrop of this transition, otoacariasis has become a problem in central hilly region of Sri Lanka.

With the changing pattern of ecology and diseases as mentioned above, it is possible that some ticks infected with rickettsial pathogens could cause otoacariasis. Many patients who present with spotted fever rickettsioses has a history of previous tick bite and it hardly produces local eschar [15, 46]. But the pathogens multiply at the tick bite site and gradually lead to systemic disease. However, asymptomatic sero-conversion among members of public has happened which denotes silent infection [47]. Therefore, it is possible that multiplication of rickettsial pathogen occur at the site of tick bite in the ear canal and subsequent direct invasion of nearby facial nerve or by local vasculitis of vasa-nervorum leaning to nerve ischaemia. As ear canal and the middle ear are enclosed in a bony structure with bony transmitters, the facial nerve remains the only soft tissue structure therein available for invading rickettsial pathogens. May be the enclosed nature of the middle ear limits the spread of the rickettsial pathogens to the rest of the body. We were able to show definitive sero-conversion against spotted fever rickettsioses in our patients in the 3rd phase of the study. This sero-conversion has happened without systemic disease similar to silent infections happening in the general public in the region. The hypothesis of rickettsial aetio-pathology is further strengthened by the direct evidence with the tick from the last patient in the series had demonstrable rickettsial DNA by qPCR together with positive qPCR with patient’s sera. Furthermore, we found prompt treatment with doxycycline making a fast recovery supporting the hypothesis. Compared to the first phase of the study where axonal degeneration was detected in NCS, those treated with doxycycline in 3rd phase had only neuropraxia suggesting that treatment has prevented progression of nerve damage. A recent study of 2000 cases of peripheral facial palsy found Bell’s palsy as the commonest, and Lyme disease was the cause in 4% of cases. Furthermore, the same study recommended adding doxycycline to treatment regimen in many situations of facial palsy.

## Conclusions

Facial palsy is a distressing morbidity that patients anticipate complete and early recovery. With rational reasoning we argued against popular toxin theory where neurotoxins in tick saliva are thought to be the cause of facial nerve palsy. Of late, we found it has a link with treatable spotted fever rickettsial infections. We consider this discovery has clinical importance as the morbidity can be minimized with the treatment with appropriate antibiotics. Therefore, we recommend the use of appropriate anti-rickettsial antibiotics along with the other supportive care in intra-aural tick related facial nerve palsies. Furthermore, tick borne infection may present with obscure clinical problems and therefore, vigilance is needed to diagnose these problems which are very often treatable.

## Abbreviations

ENT: Ear-Nose-Throat
IFT: Indirect Immuno Fluorescence Test
NCS: Nerve Conduction Studies
qPCR: quantitative Polymerase Chain Reaction
THP: Teaching Hospital, Peradeniya
